# Phenotypic plasticity and morphological integration in a marine modular invertebrate

**DOI:** 10.1186/1471-2148-7-122

**Published:** 2007-07-24

**Authors:** Juan A Sánchez, Catalina Aguilar, Daniel Dorado, Nelson Manrique

**Affiliations:** 1 Laboratorio de Biología Molecular Marina – BIOMMAR, Departamento de Ciencias Biológicas-Facultad de Ciencias, Universidad de los Andes, P.O.Box 4976, Bogotá, Colombia, USA

## Abstract

**Background:**

Colonial invertebrates such as corals exhibit nested levels of modularity, imposing a challenge to the depiction of their morphological evolution. Comparisons among diverse Caribbean gorgonian corals suggest decoupling of evolution at the polyp vs. branch/internode levels. Thus, evolutionary change in polyp form or size (the colonial module *sensu stricto*) does not imply a change in colony form (constructed of modular branches and other emergent features). This study examined the patterns of morphological integration at the intraspecific level. *Pseudopterogorgia bipinnata *(Verrill) (Octocorallia: Gorgoniidae) is a Caribbean shallow water gorgonian that can colonize most reef habitats (shallow/exposed vs. deep/protected; 1–45 m) and shows great morphological variation.

**Results:**

To characterize the genotype/environment relationship and phenotypic plasticity in *P. bipinnata*, two microsatellite loci, mitochondrial (MSH1) and nuclear (ITS) DNA sequences, and (ITS2) DGGE banding patterns were initially compared among the populations present in the coral reefs of Belize (Carrie Bow Cay), Panama (Bocas del Toro), Colombia (Cartagena) and the Bahamas (San Salvador). Despite the large and discrete differentiation of morphotypes, there was no concordant genetic variation (DGGE banding patterns) in the ITS2 genotypes from Belize, Panama and Colombia. ITS1–5.8S-ITS2 phylogenetic analysis afforded evidence for considering the species *P. kallos *(Bielschowsky) as the shallow-most morphotype of *P. bipinnata *from exposed environments. The population from Carrie Bow Cay, Belize (1–45 m) was examined to determine the phenotypic integration of modular features such as branch thickness, polyp aperture, inter-polyp distance, internode length and branch length. Third-order partial correlation coefficients suggested significant integration between polypar and colonial traits. Some features did not change at all despite 10-fold differences in other integrated features. More importantly, some colonial features showed dependence on modular features.

**Conclusion:**

Consequently, module integration in gorgonian corals can be shifted, switched or canalized along lineages. Modular marine organisms such as corals are variations on a single theme: their modules can couple or decouple, allowing them to adapt to all marine benthic environments.

## Background

Important questions in the study of evolution are whether the characteristics of organisms are mutually independent or behave and evolve as integrated systems or modules, and whether the integration of modules can be shifted, switched or canalized along lineages [1]. In modular colonial organisms, which form by the reiteration of identical units, the emergent forms are usually complex networks [2,3]. Colony form in these invertebrates is a consequence of modular (polyp) replication, and if there is tight integration (e.g. pleiotropy or linkage disequilibrium) among modular and supra-modular traits (e.g. polyp aperture, inter-polyp distance or spacing, branch thickness, internode and branch length), then changes at the module level may lead to changes in colony architecture. Alternatively, different groups of traits may change semi-independently (or conditionally independently) if pressures or developmental mechanisms differ. It has been found that all characters are mutually associated, integration being strongest among the colony level (network) characters, which suggests that branching characters within colonial organisms can be independent of module characters in interspecies comparisons [4]. Nevertheless, the integration or decomposition of modular features at the intraspecific level is unknown in marine modular organisms.

A particular attribute of gorgonian coral colonies is the existence of different levels of nested modularity, such as various types of microscopic sclerites (length variation 0.1–1 mm), polyps that always have eight pinnate tentacles (1–20 mm), and modular branches placed at nearly fixed internodes (1–50 cm), which are all repetitive modules but morphologically fixed features throughout the colony (e.g., [5-7]). Comparative analyses among several Caribbean gorgonians suggest decoupling of evolution at the polyp vs. branch levels [4]. Consequently, evolutionary change in polyp form or size does not imply a change in colony form, or vice versa. These characteristics of marine modular invertebrates are consistent with the modular concept of phenotypic plasticity widely demonstrated in plants [8]. In addition, developmental controls of colony form in gorgonian corals seem different from the traditional axial patterning of most unitary metazoans but are related to allometric changes in growth rate and reproductive onset or heterochrony [2,7]. These features of astogenetic growth also accord with the view of developmental reaction norms as whole-organism allometric functions (e.g. [9]), which reveals once again that phenotypic plasticity should be considered a feature of modular traits [8]. Could the modular concept of phenotypic plasticity explain the morphological integration of modularity in marine invertebrates such as corals?

**Figure 1:**
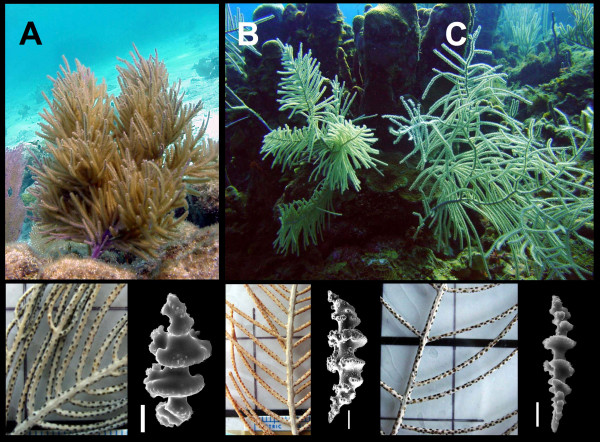
*Pseudopterogorgia bipinnata *(Verrill). A. Shallow-water bushy morphotype (= *P. kallos *[Bielchowsky]) 2 m in water depth; B. Typical regularly branched morphotype (17 m). C. Long-branched deep morphotype (17 m). Detail of dry colonies below correspond to fragments of the same colonies above (Carrie Bow Cay, Belize, 2003).

*Pseudopterogorgia bipinnata *(Verrill) (Octocorallia: Gorgoniidae) is a shallow water Caribbean gorgonian coral that can colonize most coral reef habitats. In a single location such as Carrie Bow Cay, Belize barrier reef, it is found continuously from wave-swept environments as shallow as 1 m to the deepest portions of the reef (~ 45 m) where there is very low water movement and less light (Fig. 1), as well as inshore lagoonal habitats such as sea grasses and mangrove roots in the Pelican Cays. In addition, the species ranges from purple and yellow coloration in lagoonal habitats to gray and beige in exposed fore-reef environments [9], and can adopt bushy, fan- and feather-like forms with over tenfold differences in branch length towards its environmental extremes. This explains why this species has been considered a taxonomic complex (e.g. [10,9]). To integrate findings on the evolution of modularity in gorgonian corals at the intraspecific level, the aims of this study were: (1) to ascertain, using genetic information, whether the different *P. bipinnata *"complex" phenotypes belonged to the same species; (2) to quantify the variation of different modular traits in different environments; and (3) to determine the morphological integration and decomposition of the same traits in relation to the plasticity of the species.

## Results

### Genetic differences among Pseudopterogorgia bipinnata morphotypes

**Figure 2:**
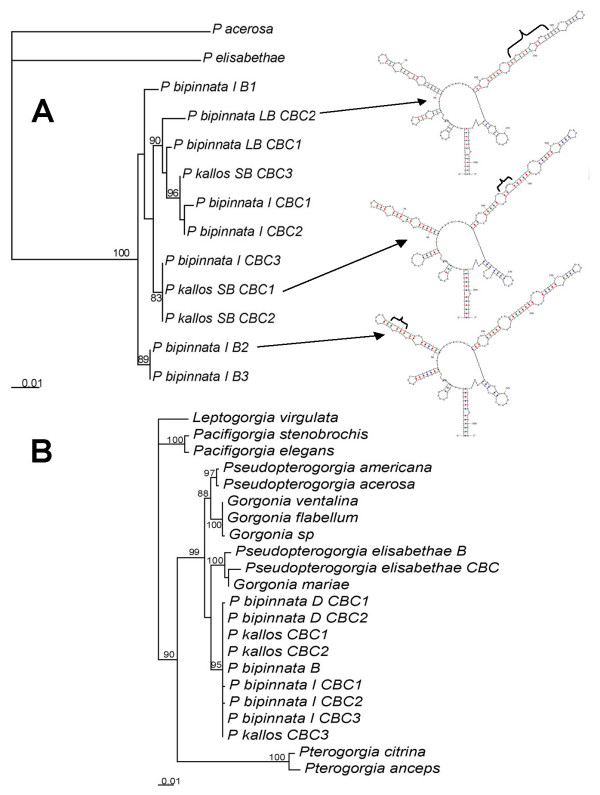
Phylogenetic hypotheses of gorgonian corals (phylograms). A. Nuclear DNA (ITS1–5.8s-ITS2, 731 bp) maximum likelihood tree using the best-fit model (TVMef+G) selected by AIC. Arrows indicate the species ITS2 secondary structure with their different morphologies. B. Mitochondrial DNA (MSH1 776 bp), maximum likelihood tree using the best-fit model (K81uf+I) in PAUP* selected by Akaike Iinformation Criterion-AIC in Modeltest. Above node support values are from 100 bootstrap replicates (>75 only). Maximum parsimony analyses yielded the same results. Scale in substitutions per site. B = Bahamas, CBC = Carrie Bow Cay, Belize, I = Typical Intermediate morphotype, D = Deep water morphotype. SEM scale = 20 μm. Additional mtDNA sequences from [4].

Mitochondrial DNA sequences from 9 colonies of the *P. bipinnata *complex were nearly invariant including 3 samples from the traditional *P. bipinnata *morphotypes from Carrie Bow Cay (CBC), Belize, here to fore referred as "typical intermediate", two of the "deep water" morphotype from CBC, one from the Bahamas (typical) and three from the species considered as *P. kallos *(Bielschowsky), smaller and bushier morphotype from shallow and exposed habitats (Fig. 1). The three morphotypes showed also slight differences in the diagnostic sclerites, surface scaphoids [10], *P. kallos *had it smaller and robust than the progressively longer and thinner of the typical intermediate and deep water *P. bipinnata *morphs (Fig. 2), though a great deal of overlapping forms were observed. The phylogenetic analyses included 14 species of the same family to show the differentiation and distance among and between other congeners of Caribbean gorgonian corals and it was clear that all the species from the complex showed no differentiation (Fig. 2A). Despite notable divergence with congeners the *P. bipinnata *"complex" showed less than 0.03 substitutions per site comparing nuclear DNA sequences (ITS1, 5.8s and ITS2: Fig. 2B). This phylogenetic hypothesis placed the three typical intermediate morphotypes from the Bahamas basally respect to 8 colonies of the three morphotypes from Belize, which were not reciprocally monophyletic. Moreover, differences where found in the ITS2 secondary structures (Fig. 2B). The two heterologous microsatellite loci showed very low variation within the *P. bipinnata *complex, most colonies had the same homozygous genotype at each locus, though a few heterozygous were observed in four colonies from the Bahamas, two from the Pelican Cays in Belize and several congeners [See additional file 1]. Compelling evidence was afforded by comparing DGGE banding patterns of the genotypes from individuals of the three morphotypes from Belize against the intermediate morphotype from Panama and the intermediate and deep morphotypes from Colombia (n = 32), no other morphotypes where found in these two locations. DGGE revealed 15 different ITS2 copies, distributed as single, double, and triple bands per individual colony, only 4 copies were shared between Belize and Panama samples and 11 copies were likely private alleles (Belize 3, Panama 4, and Colombia 4) [See Additional file 2]. Colombia had only single copy banding patterns whereas Belize and Panama had also double and triple bands in ITS2. Besides a single individual, no unique alleles where found in either intermediate or deep morphotypes, which support geographic distance as the driving force for genetic variation in ITS2 [See Additional file 2].

### Plasticity and morphologic integration in Pseudopterogorgia bipinnata

**Figure 3:**
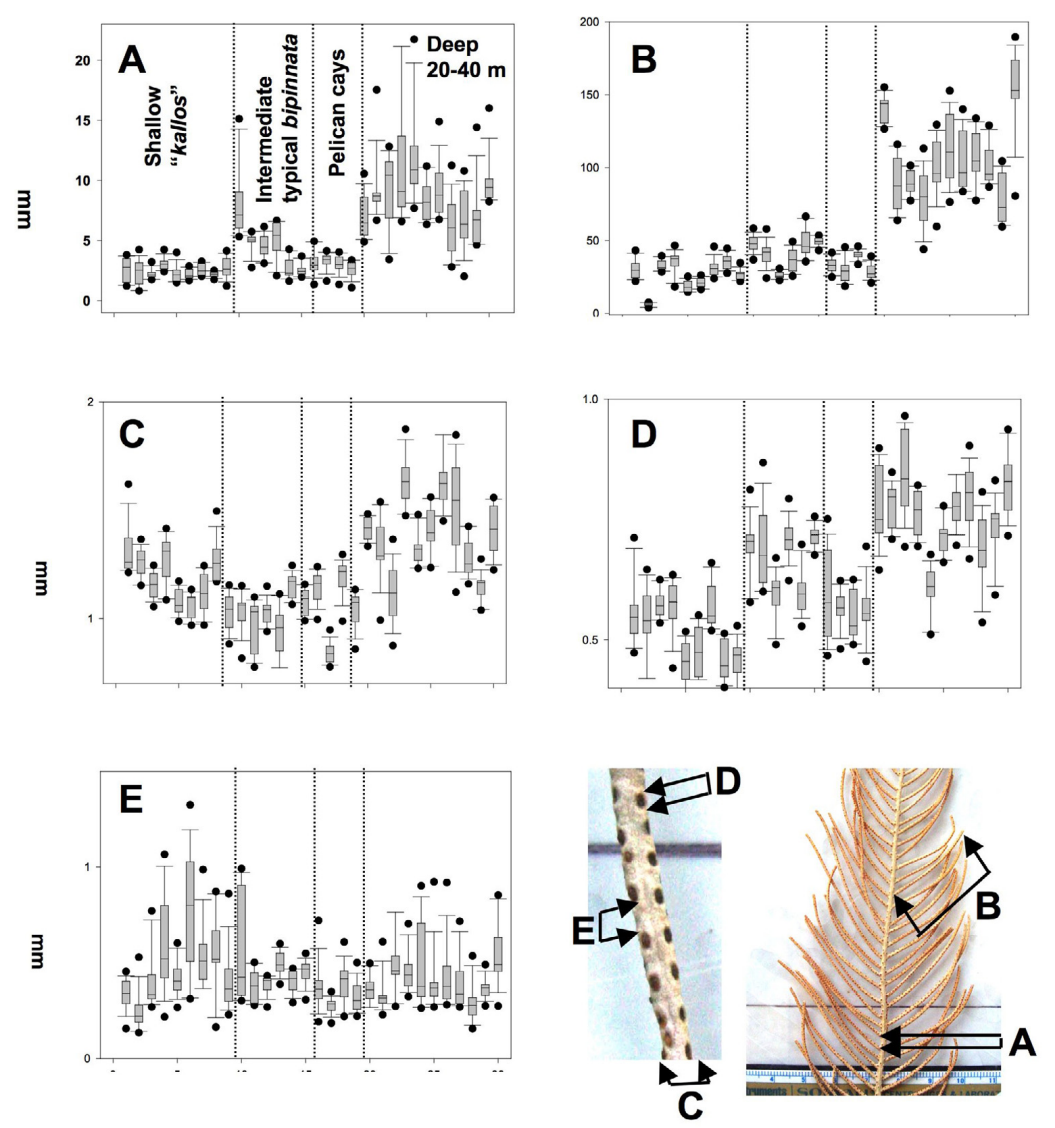
Box plots from the distribution and variance of ten measurements per colony for Branch Internode (A), Branch length (B), Branch Thickness (C), Polyp aperture (D), and Interpolyp Distance (E). The median line is inside the 25th and 75th percentiles with external error bars at the 10th and 90th percentiles.

**Figure 4:**
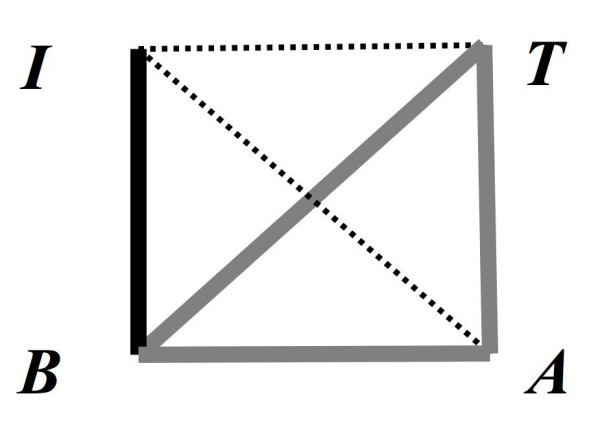
Graphical model for the morphological integration of traits in the phenotypic plasticity of *Pseudopterogorgia bipinnata*. Bold edges show strong bonds after using third-order PCC, dashed lines depict only background zero-order bivariate correlations existing among all contrast variates (Table 1). Branch Internode (I), Branch lenght (B), Branch Thickness (T) and Polyp aperture (A).

The "colonial" features branch and internode lengths were significantly longer in the deep morphotypes with a clear gap among morphotypes variances (Shallow vs. Intermediate [includes mangrove, Pelican Cays] vs. Deep; Kruskal-Wallis test, *P *< 0.001; Fig. 3A–B). There were colonies in the same reef (Carrie Bow Cay, Belize) with branches up to 13.5 times longer in the deep morphotype respect the shorter *P. kallos *branches (14–189 mm, min-max; Fig. 3B). On the other hand, the "polypar" variables had ranges between 0.4 and 2 mm. Thickness and polyp apertures were higher in the deep morphotype (K-W, *P *< 0.001) though great overlap in variances among all morphotypes occurred. Inter polyp distance was slightly higher in the shallow *P. kallos *but there were no significant differences among morphotypes (K-W test, *P *> > 0.05). Bivariate zero-order correlations, e.g., branch length vs. thickness, were all significant but those against interpolyp distance (Table 1). Third-order partial correlation coefficients (PCC), e.g., branch length vs. thickness but controlling for their correlation with the other three variables, retained only stronger correlations that suggested morphological integration. Consequently, PCC analyses revealed integrated "modules" of traits changing together across the plasticity of the *P. kallos-P. bipinnata *complex. Branch length, thickness, and polyp aperture retaining significant 3^rd ^order PCC formed an interesting loop of integration whereas internode remained alone correlating with the loop through branch length (Fig. 4).

**Table 1:** Significance levels among Third-order Partial Correlation Coefficients (PCC) for the five supra-modular variables (below the diagonal) and Bivariate (zero-order) correlation coefficients (above the diagonal). Complete name of variables: Polyp Aperture, Inter Polyp Distance, Thickness, Internode length an Branch lenght (see Fig. 3 for variable distributions and colony location)

	*Aperture*	*Distance*	*Thick*	*Internode*	*Branch*
Aperture	-		0.457*	0.75***	0.789***
Distance	n.s.	-	n.s.	n.s.	n.s.
Thickness	-0.171*	n.s.	-	0.556**	0.659***
Internode	n.s.	n.s.	n.s.	-	0.791***
Branch	0.482**	n.s.	0.442*	0.383*	-

n.s. non significant

* *P *< 0.05

**P < 0.01

*** *P *< 0.001

## Discussion

The *Pseudopterogorgia bipinnata *complex provided a good model for exploring phenotypic plasticity and morphological integration at the intraspecific level. *P. bipinnata *did not show the same pattern of morphological integration found among species, which suggests tight morphological integration of all traits and decoupling of evolution at the levels of polyp (the colonial module *sensu stricto*) vs. branch/internode (an emergent modular feature produced by colonial growth) [3]. Some features did not change at all despite 10-fold differences in other integrated features; more importantly, some colonial features showed dependence on modular features. The gorgonian corals *Pseudopterogorgia bipinnata *and *P. kallos *apparently belonged to the same species (at least phylogenetically), exhibiting remarkable plasticity, particularly in branch and internode lengths. Molecular evidence from nuclear and mitochondrial DNA sequences, as well as two microsatellite loci and ITS2 DGGE banding patterns, corroborated the view that the morphological differences corresponded to phenotypic plasticity. This finding differs from those in other corals (e.g. fire coral, *Millepora dichotoma*), where the different morphotypes found in different environments are associated with different fixed molecular patterns rather than phenotypic plasticity [11].

Allometric change during growth is a characteristic of gorgonian coral colonies. It is known that gorgonian colonies tend to spread their branches against unidirectional flow, forming a 2-dimensional network even after transplantation [12,13]. In essence, most species vary from 3-D bush-like colonies in multidirectional flows to fan-like 2-D networks in environments with unidirectional flow (e.g. [14]). Other colonial traits such as axial core composition and stiffness differ considerably within species owing to the preferred level of water movement [15]. This is related to gorgonian community structure, where many species overlap totally or partially along an environmental continuum in wave exposure and depth [9]. This change in growth forms and preferences of species may be interpreted as allometric changes that enable resources to be exploited optimally [16]. The observed change in *P. bipinnata*, in contrast, did not seem to follow a simple continuum allometric pattern but seems to support the modular concept of phenotypic plasticity [7]. An integrated allometric change that produced the nested modularity of gorgonian corals would have to include changes in most traits; but it was clear that some traits changed less than others and one trait, inter-polyp distance, did not change at all across environments.

Traits may differ in their evolutionary constraints, but the rate of change of any one trait may or may not depend on others. For gorgonians, the pattern of phenotypic integration at the intraspecific level showed the same interdependence of branches and internodes as found at the interspecific level, but also showed an integrated loop among branch length and minute "polypar" traits such as branch thickness and polyp aperture. This was not wholly inconsistent with the interspecific pattern of decoupled evolution between the "colonial" vs. "polypar" traits [3] because despite a more than tenfold change in branch and internode lengths across habitats, interpolyp distance did not correlate (e.g., zero-order PCC) with any other feature, suggesting a decoupled mechanism of change for this trait. The link between micro- and macro-evolutionary changes in the shape of modules and colonies in gorgonian corals may possibly depend on which traits are independent and which are integrated.

When a species trait is invariant in different environments while others show different phenotypic responses, there may be canalization of this particular phenotype. However, it is difficult to determine the level of canalization since different factors may interact from gene expression to morphology, and life history traits to behavior [17]. If the species *Pseudopterogorgia bipinnata *is divided in two lineages by a geographic barrier, for instance, their descendants could include short and long branched colonies that all have the same inter-polyp distance. On the other hand, inter-polyp distance might undergo a process of decanalization in which plasticity starts to occur under new environmental challenges. It is suggested that canalized traits can attain variability depending on the degree and conditions driving this event [17]. In addition, phenotypic plasticity for some traits can be an adaptive response to new selective pressures (e.g. [18,19]). The interplay between canalization and morphological integration might be the link from phenotypic plasticity to what we perceive as macroevolution.

One indication of the link between micro- and macro-evolutionary changes could be the interplay among plasticity and integration with genetic variability and assimilation (e.g., [20], but see [21]). The link between micro- and macro-evolutionary changes in the branch and polypar features of *P. bipinnata *seems to be related to constraints on change in some already-fixed features, but implies integration of changes in branch length with other unconstrained traits: inter-polyp distance, which varies widely among species [3], was constant at the intraspecific level. Moreover, other modular traits, apparently not integrated at the macroevolutionary level, became correlated with branch length at the intraspecific level (Fig. 4).

The three main phenotypes did not exhibit clines and were found in overlapping zones. Although the change of phenotype was apparently independent of genotype, two phenotypes were seldom seen side-to-side sharing the same environment (Fig. 1). This could mean that although this species has characteristic phenotypes at certain depths, there were exceptions where the ancestral, derived or intermediate phenotype could co-exist with a phenotype that was not expected to occur in these conditions. Speculatively, there could be maternal-specific developmental "signals" determining phenotype in marginal environments; or, alternatively, it could be an incipient case of genetic assimilation. It is also important to mention that phenotypic plasticity can show variations in which the population mean and variance change [22]. Therefore, the presence of two morphotypes at the same depth may just be some type of variation occurring in some locations. It would be interesting to have more estimates of these overlapping zones so that more precise conclusions could be drawn. It would be even more desirable to place experimental colonies at in-between depths to see whether there is plasticity, as seems to be the case, or whether a phenotype becomes fixed. Nonetheless, more refined molecular and experimental approaches are needed to evaluate genotypic vs. phenotypic variance.

## Conclusion

Module integration within gorgonian coral species can be shifted, switched or canalized along lineages. Marine modular organisms such as corals are variations on a single theme: their modules can couple or decouple, allowing them to adapt to all marine benthic environments. Adaptation to a certain environment does not always mean that the trait is fixed [18]. Ecotypes can make some traits exhibit phenotypic plasticity but are not so extreme as to fix these traits in the species genome (see discussion in [21]). Nonetheless, genetic assimilation [20,23] can be a viable event for octocorals, where habitat seems to be a conditioning factor for niche separation. Species that are geographically widely distributed, such as *P. bipinnata*, also tend to be widely distributed across reef habitats [9]. This trend has been found in many groups of terrestrial organisms (e.g. [24]). Even so, there is no clear evidence of such a pattern in marine organisms. Phenotypic plasticity costs have been studied and it has been shown that the type of response by the organism determines its viability (e.g. the response of enzymes to the environment has little energetic cost in *Stellaria longipes *plants: [25]; see [26] for plasticity cost models). It remains unclear whether is it better for *P. bipinnata *to fix a certain phenotype or express it depending on the environment; the costs and benefits of this plasticity have yet to be defined.

## Methods

Genetic differences among *Pseudopterogorgia bipinnata *morphotypes and congeners were initially estimated using mitochondrial and nuclear DNA sequences as well as two microsatellite loci allelic variation. Collected specimens were prepared for sclerites observations with compound microscope and a few sclerites were observed with Scanning Electron Microscopy (see methods in [27]). Mitochondrial sequences comprised the 5' end of msh1 gene using primers by [28,29] and the nuclear sequences comprised a portion of the tandem array of ribosomal genes including complete ITS1, 5.8S and ITS2 sequences using primers by [30]. DNA extractions were made according to [31] protocol using CTAB from alcohol preserved material (Ethanol 95%). PCR conditions were followed [4] and sequencing as in [32] using the Edge Biosystems kit and sequencing using BigDye 3.1 (AB 3100, capillary electrophoresis automated sequencer). The matrix editing, translation, and alignment for the diverse genes were accomplished using Bioedit [34] and ClustalW [35]. Phylogenetic analyses were carried out in PAUP* [36] coupled with Modeltest for maximum likelihood analyses [36,37]. New sequences were deposited in GENBANK (accession nos. EU04312–EU043127). Predicted RNA secondary structures were modeled for the portion corresponding to the ITS2 following the methods from [33].

Two heterologous microsatellite loci, Pel74 and Pel1, developed for the sister species *Pseudopterogorgia elisabethae *Bayer by [38] were used to assess finer genetic differences among morphotypes. For that purpose, 4 colonies from the Bahamas, 8 from Bocas del Toro (Panama), and 30 from different habitats around Carrie Bow Cay, Belize were screened. DNA extractions we made as above and PCR conditions as suggested by [38]. Microsatellite alleles were visualized using an AB3100, capillary electrophoresis automated sequencer, with fluorescently labeled primers.

In addition, a study of the ITS2 variation among morphotypes and locations was done for 32 colonies from Belize (14), Panama (12), and Colombia (6). The ITS2 region was amplified using primers and protocols from [33]. PCR reactions were separated in a Denaturing Gradient Gel Electrophoresis-DGGE (ABS) containing 8% polyacrilamide, 1× TAE Buffer and linear denaturing gradient from 40% to 80% of Urea and Formamide. Before electrophoresis the gels were pre-run at 60°C and 150 V for 20 min, followed by the electrophoresis at 60°C and 150 V for 9 h. The gels were stained with Ethidium Bromide during 15 min and analyzed using a BIORAD chemidoc system. All the reactions were conducted without CG-clamp.

Plasticity and morphologic integration in *Pseudopterogorgia bipinnata *were studied using the same traits and methods as in [3] for 30 colonies collected in the vicinity of Carrie Bow Cay, Belize, between 0.2 and 45 m of water depth (same colonies used for microsatellite loci above). Measurements (10 per colony, 30 colonies, 5 variables), included branch thickness, polyp aperture, inter polyp distance, internode and branch length. Patterns of morphological integration were examined as proposed by [1] using third-order Partial Correlation Coefficients (PCCs) that were determined among the studied variables [3].

## Authors' contributions

JAS collected all the data, did the analysis and wrote most of the manuscript. CA contributed with part of the writing and discussion as well as with the phylogenetic analyses. DD and NM did all the DGGE analyses and contributed with part of the writing. All authors read and approved the revised manuscript.

## Supplementary Material

Additional file 1 Two microsatellite loci screening. Genographer-generated Gel (ABI 3100) corresponding to two microsatellite loci: PE1 and PE74. Click here for file

Additional file 2 DGGE banding patterns. PCR-DGGE analysis of the ITS2 from *Pseudopterogorgia bipinnata *colonies from Belize (Carrie Bow Cay), Panama (Bocas del Toro), and Colombia (Cartagena). Click here for file
