# Supplementary material for: Phenotypic plasticity and morphological integration in a marine modular invertebrate
Source: BMC Evol Biol. 2007 Jul 24;7:122. doi: 10.1186/1471-2148-7-122 (PMC1959521; doi:10.1186/1471-2148-7-122)

**Additional file 1**

Two microsatellite loci screening.

Genographer-generated Gel (ABI 3100) corresponding to two microsatellite loci: PE1 and PE74.


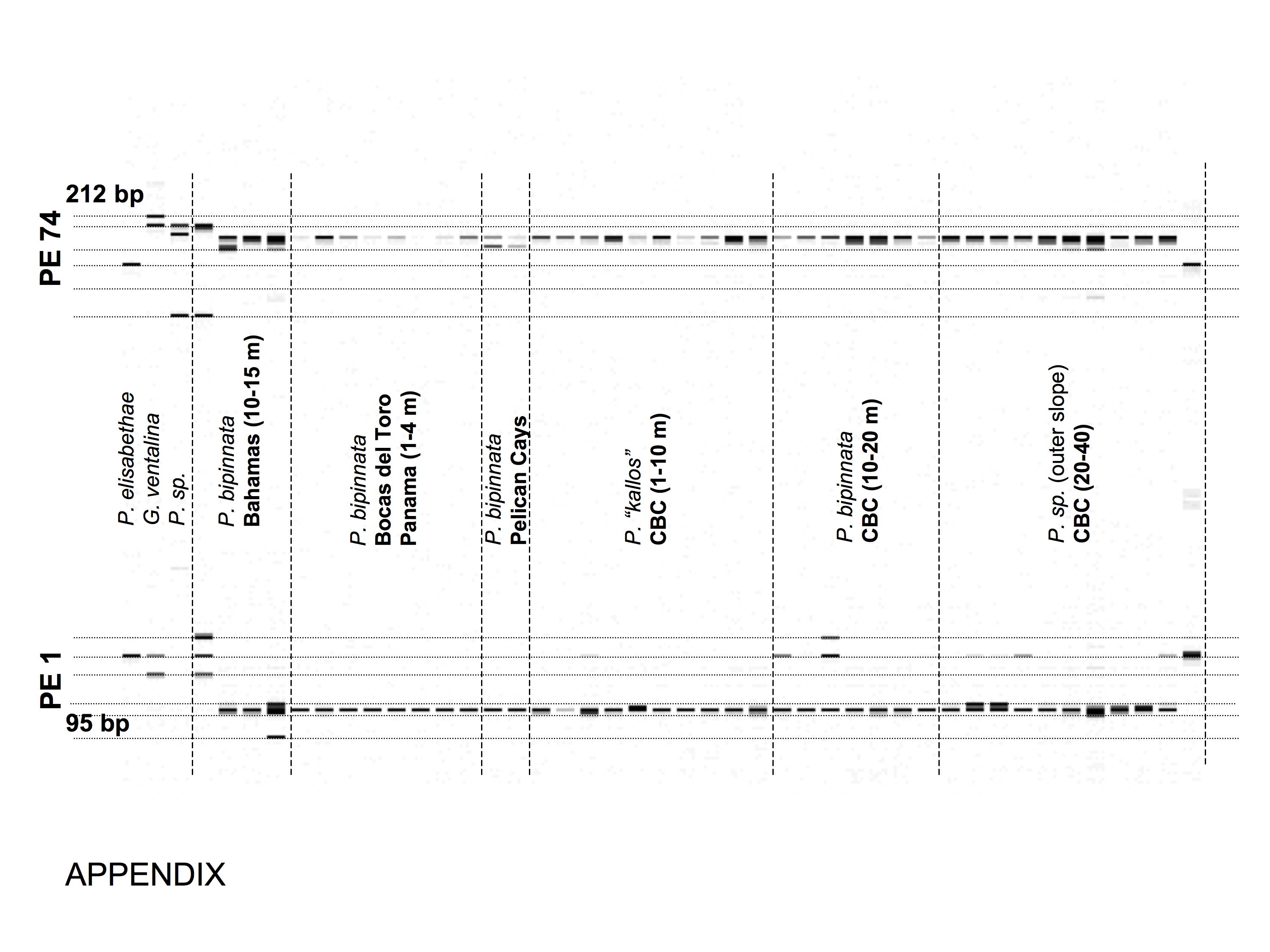

Supplement: Additional file 1 — Two microsatellite loci screening. Genographer-generated Gel (ABI 3100) corresponding to two microsatellite loci: PE1 and PE74. [file 1471-2148-7-122-S1.doc]
