# Supplementary material for: Phenotypic plasticity and morphological integration in a marine modular invertebrate
Source: BMC Evol Biol. 2007 Jul 24;7:122. doi: 10.1186/1471-2148-7-122 (PMC1959521; doi:10.1186/1471-2148-7-122)

**Additional file 2**

**DGGE banding patterns.**

PCR-DGGE analysis of the ITS2 from *Pseudopterogorgia bipinnata* colonies from A. Belize (Carrie Bow Cay), B. Panama (Bocas del Toro), and C. Colombia (Cartagena). Labels are as follows: BEL, Belize; PAN, Panama; COL, Colombia; INT, intermedium morphotype; DEE, deep morphotype. Numbers indicate putative equal ITS2 sequences.


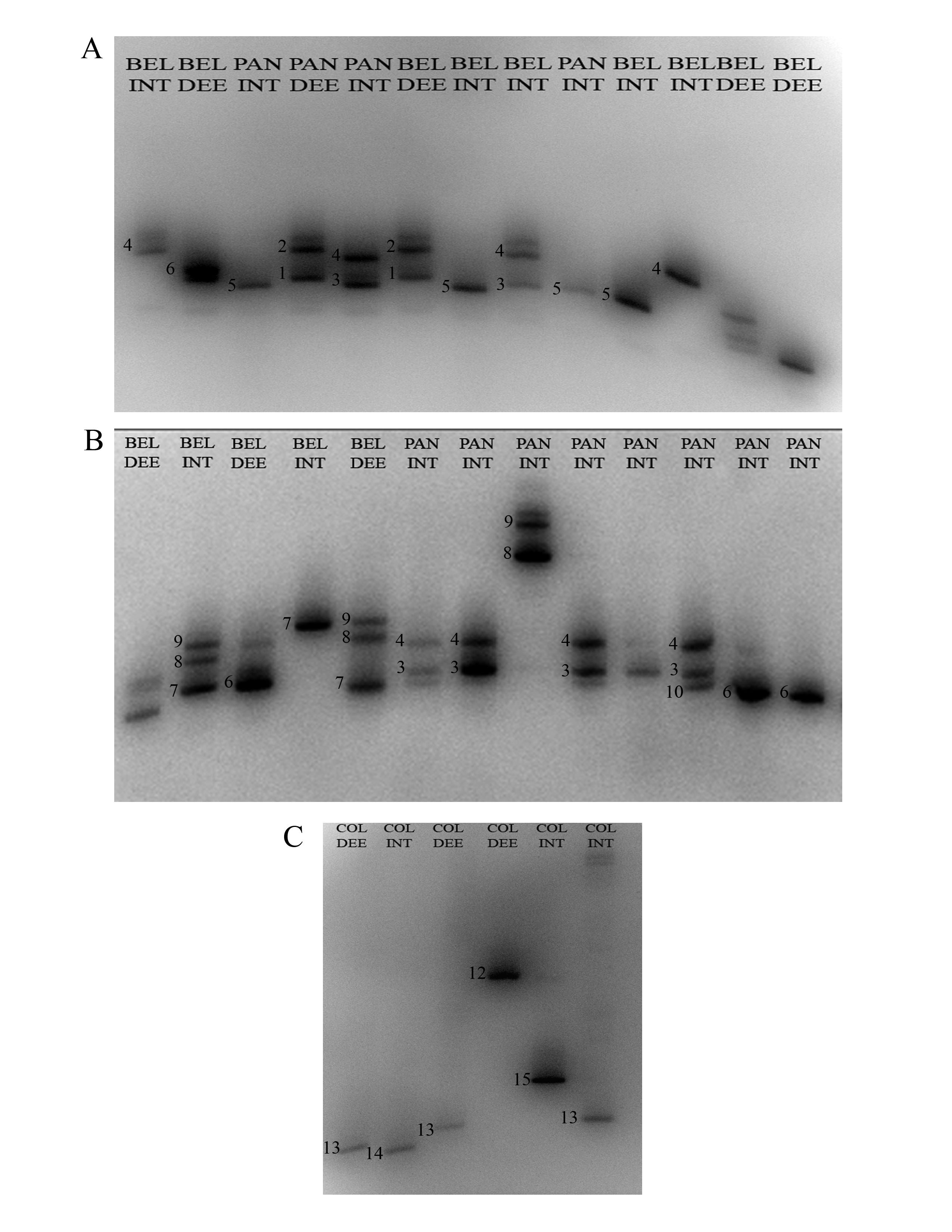

Supplement: Additional file 2 — DGGE banding patterns. PCR-DGGE analysis of the ITS2 from Pseudopterogorgia bipinnata colonies from Belize (Carrie Bow Cay), Panama (Bocas del Toro), and Colombia (Cartagena). [file 1471-2148-7-122-S2.doc]
